# A Comprehensive Review on Cyclodextrin-Based Carriers for Delivery of Chemotherapeutic Cytotoxic Anticancer Drugs

**DOI:** 10.1155/2015/198268

**Published:** 2015-10-25

**Authors:** Bina Gidwani, Amber Vyas

**Affiliations:** University Institute of Pharmacy, Pt. Ravishankar Shukla University, Raipur 492010, India

## Abstract

Most of the cytotoxic chemotherapeutic agents have poor aqueous solubility. These molecules are associated with poor physicochemical and biopharmaceutical properties, which makes the formulation difficult. An important approach in this regard is the use of combination of cyclodextrin and nanotechnology in delivery system. This paper provides an overview of limitations associated with anticancer drugs, their complexation with cyclodextrins, loading/encapsulating the complexed drugs into carriers, and various approaches used for the delivery. The present review article aims to assess the utility of cyclodextrin-based carriers like liposomes, niosomes, nanoparticles, micelles, millirods, and siRNA for delivery of antineoplastic agents. These systems based on cyclodextrin complexation and nanotechnology will camouflage the undesirable properties of drug and lead to synergistic or additive effect. Cyclodextrin-based nanotechnology seems to provide better therapeutic effect and sustain long life of healthy and recovered cells. Still, considerable study on delivery system and administration routes of cyclodextrin-based carriers is necessary with respect to their pharmacokinetics and toxicology to substantiate their safety and efficiency. In future, it would be possible to resolve the conventional and current issues associated with the development and commercialization of antineoplastic agents.

## 1. Introduction

Poor aqueous solubility and rate of dissolution are the two critical factors that affect the formulation and development process of drugs and limit their therapeutic application [1]. The administration of drugs through different route especially of those, which are poorly soluble and belong to class II or IV of biopharmaceutical classification system, represents a major challenge [2]. Also, it is remarkable that most of the cytotoxic anticancer drugs belong to the BCS class IV which comprises substances with both low solubility in aqueous fluids and low apparent permeability [3]. Although several techniques like solubilization, [4, 5] cosolvency, [6] and solid dispersion [7–9] can enhance drug's solubility, bioavailability, and dissolution properties, these methods suffer from various disadvantages such as low drug loading and large dose. As an alternative, cyclodextrin (CD) complexation came into existence and presented a great interest [10, 11]. In 21st century, the concept of utilizing dual approach (cyclodextrins and nanotechnology) has emerged as a novel plan to tackle such formulation problems [12–14]. The purpose of this review is to discuss and summarize some of the potential findings and applications of cyclodextrin-based nanocarriers for effective delivery of anticancer drugs. This paper simultaneously explores the utility of cyclodextrin complexation and nanotechnology as unique approach for development of drug delivery system. Through this system, it would be possible to move the drugs of BCS classes II and IV into class I with certain limitations.

## 2. Cyclodextrins: Types and Complexation

Cyclodextrins are chemically and physically stable macromolecules produced by enzymatic degradation of starch. They are water-soluble, biocompatible in nature with hydrophilic outer surface and lipophilic cavity. They have the shape of truncated cone or torus rather than perfect cylinder because of the chair conformation of glucopyranose unit [15].

**Figure 1:**
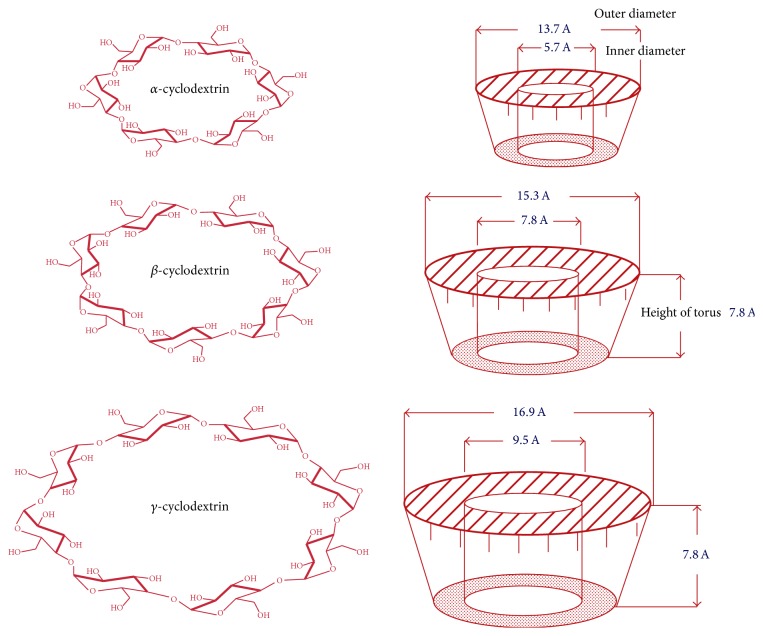
Structure and conformation of natural cyclodextrins.

Cyclodextrins are classified as natural and derived cyclodextrins. Natural cyclodextrinscomprise three well-known industrially produced (major and minor) cyclic oligosaccharides. The most common natural cyclodextrins are *α*, *β*, and *γ* consisting of 6, 7, and 8 glucopyranose units [16]. They are crystalline, homogeneous, and nonhygroscopic substances. Amongst these, *β*-cyclodextrin is ideal for complexation due to perfect cavity size, efficient drug complexation and loading, availability, and relatively low cost [17].  Figure 1 shows the structure and conformation of natural cyclodextrins. Various hydrophilic, hydrophobic, and ionic derivatives have been developed and utilized to improve the physicochemical and biopharmaceutical properties of drug and inclusion capacity of natural cyclodextrins [18–22]. Hydroxypropyl-*β*-cyclodextrin (HP-*β*-CD), randomly methylated-*β*-cyclodextrin (RM-*β*-CD), and sulfobutylether-*β*-cyclodextrin (SBE-*β*-CD) are mostly preferred for complexation [23, 24]. Polymerized cyclodextrins are high molecular weight compounds, either water-soluble or insoluble. They offer the advantage of amorphous state and complexation without toxic effects [25, 26]. The examples of polymerized cyclodextrins are soluble anionic *β*-cyclodextrin polymer, soluble *γ*-cyclodextrin polymer, and epichlorohydrin *β*-cyclodextrin polymer [27]. Due to superior solubilizing and complexing abilities exhibited these are nowadays most preferred for complexation [28, 29].  Table 1 enlists the characteristic features and properties of different types of cyclodextrins.

Inclusion complexes are formed when the “guest” molecule usually a drug is partially or fully included inside the “host's cavity” [30, 31]. Owing to the hydrophobic cavity, cyclodextrins as host offer the guest a suitable environment for interaction. The outer sphere of cyclodextrins is compatible with water, which allows hydrogen bonding cohesive interactions [32–34]. Due to this feature, CDs form inclusion complexes with a wide variety of hydrophobic compounds and change the physicochemical and biological properties of guest molecules [35–38]. These changes may enhance the therapeutic potential of drugs by diminishing their decomposition before they enter tissues and by altering how they enter tissue. The ability of a CD to form an inclusion complex is a function of steric as well as thermodynamic factors. The driving force for complexation involves the removal of water molecule from hydrophobic cavity and formation of Vander Waal forces, hydrophobic, and hydrogen bond interactions [39, 40]. The approach used for complexation is phase solubility study as described by Higuchi and Connors, which examines the effect of cyclodextrin (solubilizer/ligand) on the drug being solubilized (substrate).

## 3. Cyclodextrin Complexation with Anticancer Drugs

**Figure 2:**
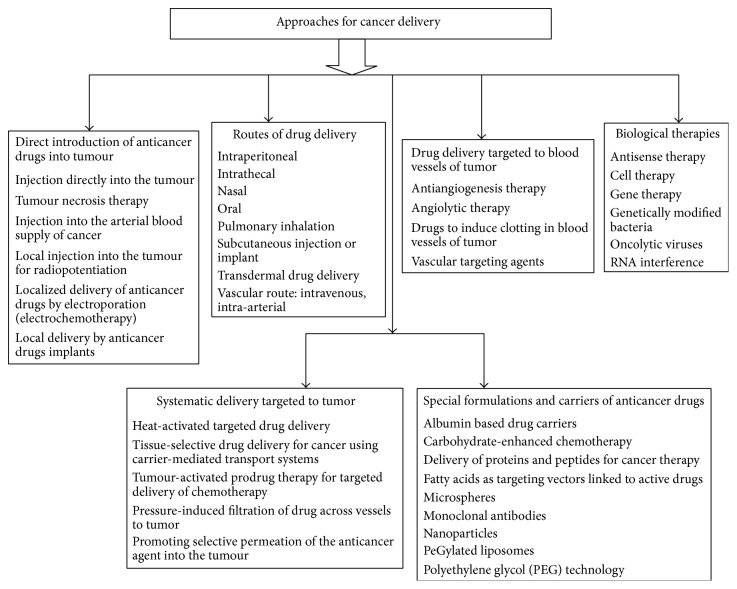
Approaches used for delivery of anticancer drugs.

Chemotherapy for cancer, particularly for recurrent and metastasis disease, has limited therapeutic effect. Limited aqueous solubility (hydrophobicity), degradation in gastrointestinal fluids, insufficient* in vitro* stability (shelf life), low bioavailability, short* in vivo* stability (half-life), affinity for intestinal and liver cytochrome P450 (CYP3A4) and P-glycoprotein (P-gp) in the intestinal barrier, poor intestinal permeabilities, and strong dose dependent side effects of promising anticancer drug candidates have long been obstacles in treatment of cancer [41]. Lack of selectivity and short blood circulation time which cause various toxic side effects are also issues of major concern [42]. The narrow therapeutic index of some anticancer drugs and the fact that these cytotoxic drugs damage not only cancer cells but also normal and healthy tissue is a major challenge. Multidrug resistance, due to increased efflux pumps such as P-glycoprotein (Pgp) in the cell membrane, which transport most of anticancer drugs out of the cell, is also major problem [43, 44]. Thus, there is a need to develop such a delivery system, which combines safety, efficacy, and convenience. Cyclodextrins are competent enough to overcome certain forms of above associated drawbacks of anticancer drugs. The lack of efficient treatment has created the need to develop and implement novel technology based on combination strategy of cyclodextrin complexation and nanotechnology with a view to make the therapy more useful and acceptable.  Figure 2 enlists the different approaches used for delivery of anticancer drugs.

The formation of inclusion complex with nontoxic agents leads to improvement in physicochemical properties of drug. Most of the anticancer drugs have been complexed with cyclodextrin and their derivatives to improve/enhance the solubility and stability, increase the bioavailability and dissolution, reduce the toxicity, and modify the physicochemical characteristics [45–57]. Complexation of doxorubicin with *γ*-CD and HP-*γ*-CD led to an increase in permeability across blood brain barrier, due to the disruption of the membrane [58]. Similarly, the *β*-CD-PEG folic acid conjugate increased the solubility of chlorambucil. Complexation of 9-nitrocamptothecin with HP-*β*-CD led to significant enhancement in antitumor activity with low toxicity [45].  Table 2 enlists the complexation of various anticancer drugs with cyclodextrins and their derivatives.

## 4. Cyclodextrin-Based Nanocarriers of Anticancer Drugs

The use of pharmaceutical carriers provides a loom, which is more time and cost-effective than new drug development [69]. Progress in nanotechnology and cellular/molecular biology has contributed to advancement in chemotherapy and gene therapy of cancer, optimistically avoiding the toxic doses of nonspecific agents. The development of new delivery system or new administration schedules offer less expensive, but more effective treatment with negligible/rare side effects [70]. Nanoparticles, with the size of about 100–10,000 times smaller than human cells, offer unique interaction with biomolecules, which may revolutionize cancer diagnosis and treatment. They have engorged surface area-volume ratio and can overcome both cellular and noncellular mechanisms of resistance, thereby increasing selectivity of drug towards cancer cell and reducing toxicity towards normal tissues [71]. Moorthi et al. described the use of nanotechnology to overcome the limitations associated with conventional cancer therapy. Some of the recent development includes the biodegradable methoxy poly (ethylene glycol)-poly (lactide) nanoparticles for controlled delivery of dacarbazine, cross-linked nanoparticles of cytarabine and microspheres of BCNU [72].

**Figure 3:**
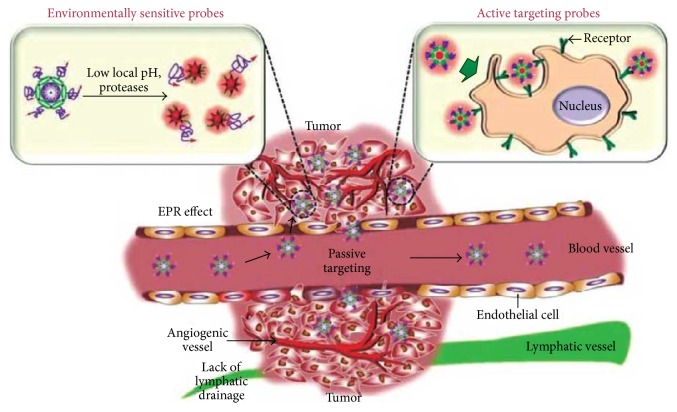
Role of EPR effect in tumor targeting.

One of the major advantages that nanotechnology offers is targeted drug delivery to the site of disease. The aim of targeted therapy is to target the chemotherapeutics drugs to cancer cell, which ultimately reduce the side effects. Active targeting or affinity targeting involves conjugation of targeting molecules (like the antibodies, ligands, and nucleic acids) on the surface of nanoparticles with receptors overexpressed on a tumor cell surface [73]. In passive targeting, liposomes, macromolecular carriers, and nanoparticles exploit the EPR effect, which is a consequence of the increased vasculature permeability and decreased lymphatic function of tumors, to target the drug to the tumor [74]. Most of the cytotoxic chemotherapeutic agents are distributed nonspecifically throughout the body and affect both the normal and tumor cells [75]. The abnormal vascular structure plays a vital role for EPR effect in tumor for targeting of certain macromolecules at the tissues. The EPR effect is different from nontargeted, passive uptake of tiny molecules.  Figure 3 shows the effect of EPR in tumor targeting. EPR is the mechanism by which a high-molecular-weight nontargeted drug/prodrug accumulates in tissues with increased vascular permeability such as in inflammatory sites and cancer. Nanotechnology based delivery system can reach tumor passively through leaky vasculature by EPR effect.

Cyclodextrin-based nanocarriers are prepared by utilizing the concept of dual approach, which involves combination of two different approaches in a single delivery system. This covers two aspects firstly; the anticancer drug is complexed with suitable cyclodextrin and secondly encapsulation of complexed drug into carrier. The encapsulation of chemotherapeutic anticancer drugs in specially designed, multifunctionalized cyclodextrin-based carriers would be a step toward their successful application in this challenging field. In drug delivery, entrapment of cyclodextrin complexed drug into nanocarriers increases the advantage of both of them. Encapsulation of cyclodextrin complexed drug into carriers will increase the drug loading capacity, entrapment efficiency, prolong the existence of the drug in systemic circulation, and reduces toxicity and provides controlled, sustained, or targeted release. Cyclodextrin adds value to the product. The unique feature of optimized drug-cyclodextrin complex includes lower aggregation; better ADME properties, rare toxicity, and are patient friendly [76].

Safety is important criteria for consideration before using cyclodextrins as pharmaceutical excipients. The safety and toxicity of cyclodextrins depend on the route of administration. When administered orally, cyclodextrins are not absorbed from gastrointestinal tract and thus are practically nontoxic. This is due to their bulky and hydrophilic nature. Any absorption, if it occurs, is by passive diffusion. Most of the hydrophilic derivatives of natural cyclodextrins like 2-hydroxypropyl-*β*-cyclodextrin and sulphobutylether-*β*-cyclodextrin are considered safe for parenteral administration. Higher dose of cyclodextrins may be harmful. Parenterally (intravenous) administered CDs disappear rapidly from systemic circulation and are renally excreted intact. When administered, CDs are distributed to the kidney, liver, urinary bladder, and various other tissues of the body. As per toxicity, profile of cyclodextrins is considered; several* in vitro* studies have reported the hemolytic effects of CDs although the toxicological implication of* in vivo* study is considered negligible. In 2008, Stella and He discussed the detailed safety and toxicity aspects of cyclodextrins with suitable examples [77].

Considering the regulatory aspects of various cyclodextrins, it is still evolving. Monograph of parent cyclodextrins (*α* and *β*) is available in various Pharmacopoeias like US Pharmacopeia, European Pharmacopeia, Japanese Pharmacopeia, and National Formulary. The monographs for various hydrophilic derivatives of cyclodextrins are also included in compendial sources like The Handbook of Pharmaceutical Excipients. Efforts are under process for inclusion of all other cyclodextrins in this list. Natural cyclodextrins are also included in US FDA list generally regarded as safe for their use as food additives [78]. The hunt for proficient and safe carrier to attain better drug availability at the targeted site has been an exigent area of research. Encapsulation of antineoplastic agents in carrier vesicles led to decrease in drug induced toxic side effects and increase in antitumor efficacy. Some of the cyclodextrin-based carriers of anticancer drugs are discussed with examples.

### 4.1. Cyclodextrin-Based Liposomes

Liposomes are concentric vesicles in which an aqueous volume is enclosed by membranous lipid bilayer. They entrap hydrophilic drug in the aqueous phase and hydrophobic drug in the lipid bilayer and retain drugs in route to their destination. In liposomes, cyclodextrin complexation competes with liposomal membrane binding, which tempers the potential benefit of complexation in prolonging hydrophobic drug retention [79]. The entrapment of water-soluble cyclodextrin-drug inclusion complexes in liposomes leads to accommodation of insoluble drugs in the aqueous phase of vesicles, increases the drug to lipid ratio, enlarges the range of encapsulation, allows targeting of complexes to specific sites, and reduces toxicity. Some of the examples are discussed as under Arima et al., in the year 2006, examined the antitumor effect of PEGylated liposomes of DOX complexed with *γ*-CD, administered through intravenous injection in BALB/c mice bearing colon-26 tumor cells. Results reflected retardation in tumor growth, increase in drug retention, and improvement in survival rate [59]. Dhule et al. 2012 evaluated the liposomal curcumin's potential against cancer models of mesenchymal (OS) and epithelial origin (breast cancer). The 2-HP-*γ*-CD/curcumin-liposome complex showed promising anticancer potential both* in vitro* and* in vivo*. In another study, the antiproliferative and cytotoxic activity of anticancer agent LPSF/AC04 in cyclodextrin complexed liposomes was enhanced [60]. Cui et al. 2011 developed stable PEGylated liposomal vincristine formulation with enhanced efficiency using sulfobutyl ether cyclodextrin as trapping agent. This formulation prolonged the circulation half-life from 43.6 to 70.0 hrs and reduced toxicity [80]. Thus, this strategy can incorporate several other cyclodextrin complexed anticancer drugs into liposomes and improve their retention inside vesicles.

### 4.2. Cyclodextrin-Based Niosomes

Niosomes are spherical lipid bilayers that can entrap water-soluble solutes within aqueous domains or alternatively lipid molecules within the lipid bilayers. They are prepared by hydrating the mixture of cholesterol and nonionic surfactants. These are vesicular systems similar to liposomes, biodegradable, biocompatible, and nonimmunogenic in nature and exhibit flexibility in their structural characterization. They are preferred over liposomes due to the higher chemical stability and economy. They alter the tissue distribution, cellular drug interaction and plasma clearance kinetics of the drug [81]. Oommen et al. 1999 entrapped methotrexate (MTX) complexed with *β*-cyclodextrin into niosomes. Complexation increased the entrapment efficiency and improved the anticancer activity. The entrapment efficiency was higher in the case of niosomes of MTX-*β*-CD complex (84%) compared to plain drug (67%). A relatively slow drug release of entrapped drug-complex from the vesicles compared to plain MTX encapsulated niosomes was observed [61]. This approach can manage the duration of action, in those cases where the dissociation constants of inclusion complexes can be modified.

### 4.3. Cyclodextrin-Based Nanosponges

Nanosponges are a class of microscopic particles with cavities of few nanometers wide, characterized by the capacity to encapsulate a large variety of substances that can be transported through aqueous media. Cyclodextrin-based nanosponges (NS) are prepared by cross-linking cyclodextrins (CD) with a carbonyl or a dicarboxylate compound as cross-linker. They are solid particles with spherical morphology with very high solubilizing effect and forms inclusion and noninclusion complexes with various drugs. The CD-cross-linker ratio can be varied to improve the drug loading and obtain a tailored release profile [82]. Swaminathan et al. 2010 prepared cyclodextrin-based nanosponges encapsulating camptothecin, for prolonging the shelf life and drug release. Camptothecin (CAM) has limited therapeutic utility because of its poor solubility, lactone ring instability, and serious side effects. The zeta potentials of cyclodextrin-based nanosponges of camptothecin were sufficiently high (−20 to −25 mV) leading to stable colloidal nanosuspension [62]. The* in vitro* studies indicated slow and prolonged drug release over 24 hrs and the cytotoxicity study showed that the formulations containing CAM were more cytotoxic than pure CAM. Mognetti et al. 2012 prepared paclitaxel-loaded *β*-cyclodextrin nanosponges, a water stable colloidal system avoiding the recrystallization of paclitaxel. The* in vitro* release studies showed that complete drug release was obtained within 2 hrs without an initial burst effect. The delivery of paclitaxel via nanosponges increased the amount of paclitaxel entering cancer cells and lowered the paclitaxel IC50, thereby enhancing its pharmacological effect [63].

### 4.4. Cyclodextrin-Based Micelles

Micelles are self-assembled nanosized colloidal particles with lipophilic central part and hydrophilic covering. They have a single, central, and primarily hydrophobic zone or “core” surrounded by a hydrophilic layer or “shell.” They have size in the range of about 5 to 2000 nm. They can entrap hydrophobic drugs at the core, which are transported at concentrations exceeding their intrinsic water solubility. The hydrophilic periphery of the micelle renders the polymer water-soluble and provides a tight shell around the drug-loaded core. This minimizes drug degradation and harmful side effects but increases bioavailability. Thus, these micelles can give a better therapeutic profile [83]. Drug is encapsulated into polymeric micelles either covalently or by physical encapsulation. Cyclodextrins as host are exploited for designing of self-assembled networks called polymeric micelles with guest molecules. A cyclodextrin micelle (CDM) contains derivatives of cyclodextrin including dimers, trimers, and polymers, incorporated as amphiphilic molecules and forms aggregate. Liu et al. 2012 formulated multifunctional pH-disintegrable micellar nanoparticles fabricated from asymmetrically functionalized *β*-cyclodextrin-based star copolymers covalently conjugated with doxorubicin (DOX), folic acid (FA), and DOTA-Gd moieties for targeted drug delivery. Results showed enhancement in drug release due to acid-labile feature of carbamate linkage [64].

### 4.5. Cyclodextrin-Based Polymeric Millirods

Site-specific, controlled release of cytotoxic agents from biodegradable polymer depots is a rising trend involved in cancer chemotherapy. Drug inclusion in a polymer depot also allows for potential tailoring of release kinetics, adding the benefit of being able to design the most efficacious delivery regimen. The incorporation of cyclodextrins into polymer millirods for complexing drugs significantly improves the drug release kinetics with various release patterns. The millirod consists of two functional compartments: an inner drug-loaded monolithic millirod as the drug depot and an outer NaCl-impregnated polymer membrane to control the release rate of the drug. The inner part of millirod permits the entrapment of drug particles in the matrix and provides sustained drug release [84]. Wang et al. 2006 prepared PLGA polymeric millirods for local delivery of *β*-lapachone. Complexation with HP-*β*-CD prevented the dissolution of drug and led to fast release (approximately 80%) after 2 days [65]. This data demonstrated the ability to tailor release kinetics via CD complexation and provided exciting opportunities for the use of millirods in intratumoral drug delivery.

### 4.6. Cyclodextrin-Based Nanoparticles

**Figure 4:**
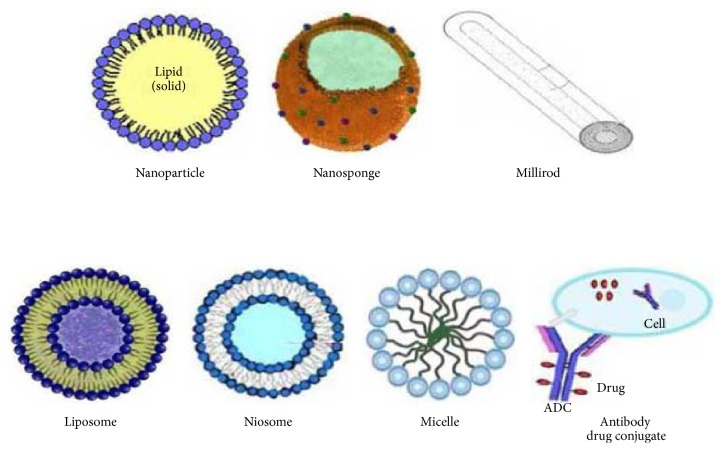
Cyclodextrin-based carriers for delivery of anticancer drugs.

Nanoparticles are solid colloidal particles compos6ed of natural, synthetic, or semisynthetic polymers with size range from 1 nm to 1000 nm. Cyclodextrins increase the loading capacity of nanoparticles. Çirpanli et al. 2009 reported that the release of camptothecin was extended to 12 days with amphiphilic *β*-CD nanoparticles and 48 hrs with polymeric nanoparticles, showing the superiority of CD based nanoparticles over conventional polymeric nanoparticles [66]. The anticancer efficacy of amphiphilic CD nanoparticles was higher than that of PLGA/PCL nanoparticles loaded with CPT and its solution in DMSO. Agüeros et al. 2009 reported that the zeta potentials of the colyophilized nanoparticles of cyclodextrin complexed paclitaxel indicated stable colloidal dispersions within the range of −18 to −39 mV. Thus, paclitaxel encapsulation was higher with threefold increase in loading capacity [67]. Quaglia et al. 2009 prepared nanoparticles of the amphiphilic cyclodextrin heptakis (2-Ooligo (ethyleneoxide)-6-hexadecylthio-)-*β*-CD (SC16OH) entrapping docetaxel (Doc) to achieve prolonged drug release [68].  Table 3 covers the cyclodextrin-based nanocarriers for most of the anticancer drugs.  Figure 4 shows the cyclodextrin-based carriers used in delivery of anticancer drugs.

### 4.7. Cyclodextrin Grafted Polymeric Nanocarriers

In this type of delivery system, natural cyclodextrins are grafted with polymers and then utilized for complexation with drugs. These complexes are then loaded into nanocarriers. Grafting is done by conjugating several units of cyclodextrins on polymer, to increase the binding ability of guest molecules and scale up the production. These polymers made of organized CDs provide the opportunity for drug molecule to associate at different levels within the nanostructure. For example, Zhang et al. 2011 designed a *β*-CD functionalized hyper branched polyglycerol (HPG-*β*-CD) of paclitaxel to achieve high drug loading capacity and aqueous solubility. The prepared nanoparticles had good biocompatibility and proved to be promising delivery system for hydrophobic drugs [85]. Recently, Zeng et al. 2013 prepared hollow nanospheres of camptothecin complexed with *β*-CD-graft-PAsp (*β*-cyclodextrin grafted with poly aspartic acid). Complexation led to improvement in aqueous solubility and stability of CPT. The nanoassemblies (nanospheres) of CPT-*β*-CD-graft-PAsp led to passive targeting of drug, sustained release, and decreased cytotoxicity [86]. These proved that cyclodextrin grafted polymeric nanocarriers can provide multifunctional properties for effective delivery of anticancer drugs.

### 4.8. Cyclodextrins Based Magnetic Nanoparticles

Cyclodextrins based magnetic colloidal nanoparticles are developed and fabricated to achieve targeted delivery of hydrophobic anticancer drugs. This is done by conjugation or grafting cyclodextrins with the drug through a linker moiety. For example, Badruddoza et al. in 2013 synthesized uniform nanocomposite for cell targeting of hydrophobic compounds. The magnetic particle Fe_3_O_4_ was encapsulated within the shell of silicon-dioxide using folic acid as the cell targeting ligand and *β*-cyclodextrin as the vehicle. These theranostic nanocomposite magnetic particles possessed multifunctional properties like cell targeting, fluorescence imaging, and delayed release [87]. Recently Sahu and Mohapatra in the year 2013 developed multifunctional magnetic fluorescent hybrid nanocarrier of 5-fluorouracil. Complexation led to increase in aqueous solubility and fluorescent carrier led to increase in stability and magnetic delivery of drug [88]. Several other hydrophobic anticancer drugs like curcumin [89], all-trans-retinoic acid, and methotrexate were also delivered using the same approach. In future, the concept of utilizing this approach of controlled release and drug targeting technology could provide more efficient and less harmful solution to conquer the limitations associated with conventional chemotherapy.

### 4.9. Cyclodextrin-Based siRNA (Short Interfering RNA) Delivery System

Small pieces of nucleic acid, known as siRNA, are one of the promising carriers for delivery of anticancer drugs. Free siRNA do not produce efficient and predictable therapeutic effect. They have biological half-life of less than an hour in human plasma. Thus, to increase the life span and improve the therapeutic efficacy, nonviral vectors are used. The therapy based on cyclodextrins and siRNA is currently under investigation for the treatment of cancer [90]. Synergistic therapeutic effect and regulation in tumor pathways are achieved by combining the drug with orthogonal therapeutic moieties like siRNA. Kim et al. 2011 synthesized cyclodextrin-modified dendritic polyamines for translocating siRNA and anticancer drugs suberoylanilide hydroxamic acid and erlotinib. The presence of *β*-cyclodextrins facilitated complexation and intracellular uptake of hydrophobic anticancer drugs, whereas the cationic polyamine backbone allowed electrostatic interaction with the negatively charged siRNA. Codelivery of siRNA-EGFRvIII and SAHA/erlotinib in glioblastoma cells significantly inhibited cell proliferation and induced apoptosis compared to the individual treatments. Also, the DexAM complex possessed minimal cytotoxicity over a wide range of concentration and efficiently delivered siRNA, thereby silencing the expression of targeted genes. Thus, this study led to synergistic induction of apoptosis in brain cancer cells by targeted codelivery of siRNA and anticancer drugs [91]. Deng et al. 2011 synthesized star-shaped polymers for sustained delivery of methotrexate. This polymer exhibited higher transfection efficiency with low cytotoxicity in fibroblast cells. *β*-cyclodextrin was used simultaneously for the entrapment and sustained release of methotrexate [92]. Another important contribution in this field is the cyclodextrin-based nanoparticles designed to deliver siRNA agent to reduce the production of ribonucleotide reductase subunit M2 (RRM2). The study addresses the relevance of siRNA nanoparticles delivery for tumor-specific targeting. A/J mice bearing subcutaneous Neuro2A tumors were treated by intravenous injection of siRNA-containing nanoparticles formed with cyclodextrin-containing polycations (CDP). Results revealed that transferrin- (Tf-) targeted nanoparticles containing two different siRNA sequences slowed the tumor growth, whereas the nontargeted nanoparticles were considerably less efficient, when treated at the same dose. Another anticancer agent CALAA01, a targeted, self-assembling nanoparticles system based on CD complexed siRNA has been effective in phase I clinical trials for the treatment of solid tumors [93]. These examples highlight the recent advances in development of nanoparticles based on linear and cyclodextrin-based polymers for the treatment of cancer.

### 4.10. Cyclodextrin-Based Monoclonal Antibody Drug-Conjugate Approach

Many cytotoxic chemotherapeutic drugs have failed in clinical trials due to their extreme toxicity and lack of satisfactory therapeutic activity at the maximal tolerated dose (MTD). An important aspect of improving therapeutic activity of these anticancer drugs is to conjugate them with antibodies. Antibodies recognize the tumor-associated, cell surface antigens. Monoclonal antibodies (mAbs) represent a major class of agents currently used for cancer treatment. Therapeutic mAbs display better pharmacokinetic parameters, with moderate or no systemic toxicity [94]. The cytotoxic drugs covalently linked to a monoclonal antibody that recognizes a tumor-associated antigen is the antibody drug conjugate. This conjugate combines the selectivity, favorable pharmacokinetics, biodistribution, and functional activity of antibodies with the high cytotoxic potency of drug. A novel contribution in this regard is the antibody drug conjugate (ADC) of trastuzumab. Trastuzumab emtansine (T-DM1) is an ADC consisting of the anti-HER2 mAb trastuzumab (Herceptin) and the maytansinoid DM1 has been administered safely at therapeutically effective doses, despite HER2 being expressed on some normal tissues [95–98]. Genentech/Roche and their team are currently developing the ADC T-DM1 for patients with advanced HER2-positive breast cancer who have previously received multiple HER2-targeted medicines and chemotherapies [99]. Only few antibodies, tested against various tumor-associated antigens, such as rituximab, cetuximab, and panitumumab, are into the clinical practice [100]. Currently, researchers are interested in targeting of cancer stem cell specific antigens with antibody drug conjugate. The unique feature of monoclonal antibody drug conjugate is that it can overcome or minimize the multidrug resistance (MDR), which is a major obstacle in successful chemotherapy [101]. Thus, monoclonal antibody drug conjugate technology can deliver the powerful cytotoxic anticancer drugs with improved/enhanced pharmacokinetic properties. Earlier in 2003 Ikura et al. prepared A7 monoclonal antibody to *β*-CD conjugate using cell fusion technique, which served as useful tool for detection of *β*-cyclodextrin and its derivatives both quantitatively and qualitatively with its applications in various fields [102]. In 2003, Johns et al. investigated the antitumor efficiency of cytotoxic drugs in combination with (monoclonal antibody) mAb 806 by EGFR (epidermal growth factor receptor) inhibitor AG 1478. AG1478 is an inhibitor of EGFR tyrosine kinase, widely used in the laboratories [103]. However, it is insoluble in water and its various therapeutic potential is under process. Sulphobutyl ether *β*-cyclodextrin was used to solubilize AG1478. The investigation of John et al., 2003 proved that combination of cytotoxic drug with mAb can provide synergistic effect and enhanced antitumor efficiency. In future, this approach could be utilized for treatment of tumors.

### 4.11. Cyclodextrins Based Supramolecular Vesicles

Supramolecular complexes are formed by the combination of cyclic molecules with polymers (linear and/or branched). The use of supramolecular vesicles in drug delivery is expanding tremendously [104]. In case of cyclodextrin-based supramolecular complexes, the cyclic molecule is natural cyclodextrins and the polymers are PVA, PEG, PTA, PBA, PPG, and so forth; moreover, when the guest moiety (drug) is attached covalently to a host molecule (cyclodextrin) in a suitable way, an intra/intermolecular complex is obtained from supramolecular oligomer and polymer. Recently, the supramolecular self-assembly of cyclodextrins and polymers has led to the development of novel supramolecular hydrogels for drug delivery applications [105]. Nowadays supramolecular vesicles are developed for delivery of anticancer drugs. For example, a supramolecular linear-dendritic copolymeric micellar carrier of paclitaxel was developed using polystyrene and hyperbranched polyglycerols along with *β*-cyclodextrin [106]. This system provided higher loading capacity of paclitaxel. In another study, Paolino et al. 2012 investigated the potential of folate targeted supramolecular vesicular aggregate of GEM for treatment of solid tumors and breast cancer through* in vivo* models [107]. Thus, the supramolecular vesicles based delivery system could represent a novel approach by using self-assembling carriers and biocompatible polymers as potential for treatment of cancer.

## 5. Formulation Containing Cyclodextrin

An ideal drug delivery system should deliver the required amount of drug to the targeted site both efficiently and precisely, for a desired time. For a drug molecule to be pharmacologically active, it must have some amount of aqueous solubility and lipophilicity in order to permeate the biological membranes through passive diffusion. The potency (effectiveness) and type of formulation determine the aqueous solubility of any drug [108]. A hydrophilic drug will not be able to partition from the aqueous exterior layer into lipophilic biomembrane. Improved therapeutic efficiency and targeted delivery of preexisting and new drugs can be achieved through carrier based novel drug delivery system, which is most suitable and approachable method for development of delivery system. Oral delivery of cytotoxic anticancer agents eliminates/minimizes the need for hospitalization, medical assistance, and infusion equipment. Cyclodextrins are used for encapsulation of lipophilic drugs and have demonstrated the ability to inhibit both PGP and cytochrome P450 localized on the surface of enterocytes [109]. To exploit the useful properties of both these above-mentioned features one of the anticancer drugs, PTX (paclitaxel) was loaded into poly (anhydride) nanoparticles after complexing with cyclodextrins. Interestingly, the relative oral bioavailability of PTX-cyclodextrin complex was 80%, which is remarkable for oral formulations. This example shows the promising use of cyclodextrins in oral formulations for delivery of anticancer drugs [110].

Cyclodextrins (CDs) form inclusion complexes with many drugs by trapping the molecule or part of it into the hydrophobic cavity. They are used as formulation additives and transdermal absorption promoters in topical delivery. Bilensoy et al. 2007 prepared vaginal gel formulation loaded with cyclodextrin complexed 5-fluorouracil, having thermosensitive and mucoadhesive properties. This ensures the longer residence of gel at the vagina, HPV-infection site, and the genital tract. Complexation provided favorable drug release with the reduction in side effects. This could be an efficient therapy for HPV-related diseases such as cervical cancer or genital warts with a lower dose.

Injectable formulations of water-insoluble drugs mainly consist of mixture of water, organic cosolvents, and surfactants. The use of organic solvents led to drug precipitation and cause pain, inflammation, and haemolysis [111]. Isotonic aqueous solution of cyclodextrins can replace the use of organic solvents and surfactants in injectable formulations. Among various cyclodextrins, HP-*β*-CD and SBE-*β*-CDs due to their high aqueous solubility and minimum toxicity are widely used in parenteral delivery. The advantage of using CDs in parenteral formulation includes solubilization of drug, reduction of drug irritation at the site of administration and stabilization of drugs unstable in aqueous environment, and so forth [112]. The use of cyclodextrins can reduce* in situ* irritation resulting from direct chemical irritancy of drugs, which cause phlebitis and pain at the site of injection. After intravenous injection, the drug is released rapidly and quantitatively from the complex upon dilution, followed by competitive replacement and binding to tissue and plasma proteins. Cyclodextrin exerts no effect on the pharmacokinetics of injected drugs. Ma et al. 1999 prepared injectable formulation of melphalan with SBE-*β*-CD and HP-*β*-CD. Through this formulation, the shelf life, solubility, and stability of the reconstituted melphalan were enhanced. Further, Oomen et al. 2010 prepared niosomal formulation for subcutaneous delivery of plumbagin. Complexation with *β*-cyclodextrin led to increase in aqueous solubility, stability, and efficacy and the niosome-entrapped drug-complex had improved anticancer activity as evidenced by the enhanced volume doubling time and growth delay [113]. Li et al. 2011 investigated the single and repeated-dose pharmacokinetics of injectable *β*-cyclodextrin-oridonin inclusion complex in rats. The results showed significant increase in the solubility and bioavailability of oridonin in rats. Finally, the hydrophilic cyclodextrin derivatives like hydroxypropyl-*β*-cyclodextrin and sulfobutylether *β*-cyclodextrin are relatively nontoxic and have minimal effect on the intrinsic pharmacokinetics of drugs. Thus, the formulations containing cyclodextrin are progressively being used during* in vitro* and* in vivo* screening of new anticancer compounds/drugs.

## 6. Current Status of Cyclodextrin

The impact of cyclodextrin-based nanocarriers and their therapeutics will likely accelerate in coming years. However, as these products move out of the laboratory and into the clinics, various federal agencies like FDA and US patents have to struggle in order to encourage the development of these products. This section highlights the contribution of those cyclodextrin-based therapeutic systems, which are under clinical trials or have been approved for human use [114]. Recently FDA approved the liposomal preparations of doxorubicin (Doxil), daunorubicin (DaunoXome), cytarabine (DepoCyt), and amphotericin B (Abelcet) which have proven to be attractive and less toxic alternatives to the conventional drug formulations and have opened new hopes for researchers. Until now, five (passively targeted) micelle products for anticancer therapy have been investigated in clinical trials, of which one has been granted FDA approval (Genexol-PM) for its use in patients with breast cancer [114]. Several liposomal formulations of conventional anticancer drugs are currently in phase I/II evaluation, including liposomal vincristine, platinum, mitoxantrone, all-trans-retinoic acid, and lurtotecan. There is a strong probability that these drug carriers will allow better administration of poorly soluble cancer drugs, enhance drug delivery and uptake in the tumor, and boost dose intensity, subsequently improving antitumor response. These recent reports favor the need of novel systems in this field.

In past 5 years, the significant contributions of cyclodextrins and their derivatives in drug delivery are discussed here, Dabur Pharma, one of India's leading manufacturers of anticancer drugs, launched Nanoxel, a novel drug delivery system for Paclitaxel, in 2007. This nanoscale drug delivery system is India's first indigenously developed nanotechnology-based chemotherapeutic agent throwing open a larger window for antitumor activity. In addition, the market for cyclodextrin-based drug delivery is remarkably increasing. Taj Pharmaceuticals (Mumbai based generic manufacturing Indian Pharmaceuticals Company) announced FDA approval for manufacturing of Piroxicam-beta-Cyclodextrin, Nimesulide-beta-cyclodextrin, Aceclofenac-beta-Cyclodextrin generic drugs in regulatory market, and nonregulated market in the year 2009. The use of sulphobutylether-*β*-cyclodextrin is tremendously increasing in formulation and development. Recently FDA has approved five drug products containing captisol. They are Vfend I.V. Solution containing voriconazole, used in treatment of fungal infections, Nexterone containing amiodarone, used in ventricular arrhythmia, Geodon containing ziprasidone, used in schizophrenia, and so forth. This shows the wide use of cyclodextrins in pharmaceuticals. The number of the cyclodextrins-containing pharmaceutical products approved and marketed has been continuously increasing. Worldwide there are about 40 products or formulations containing various CDs, especially *β*-CD and its derivative. However, till date there is no single formulation containing anticancer drug with cyclodextrins in the market. This shows that there is a need to exploit the utility of cyclodextrins in the development of delivery system for anticancer drugs in the coming years.

## 7. Conclusion

Various nanotherapeutic approaches have been developed for delivery of anticancer drugs. But, still none of the available treatments for cancer is safe, effective, and able to treat the disease completely. Most of these are expensive, unacceptable, and inconvenient for long-term use or associated with significant toxicity. Thus, there is a need to move a step forward and utilize an advanced technology based on combination strategy, which exploits the advantages of both the systems, namely, cyclodextrin complexation and nanotechnology in single delivery system. The use of drug delivery systems, based on colloidal vesicles and macromolecular carriers (cyclodextrins), represents a promising and innovative strategy that enables effective therapy with minimum side effects. However, the major challenge is about the toxicity and pharmacokinetic study of these cyclodextrin-based carriers. Most of the information about toxicity is based on* in vitro* cell models. There are several studies concerning the interaction of nanoparticles within the body. But, there is no significant result for interaction of cyclodextrin complexed nanoparticles in the body. Also, there is a need to throw light onto the route of administration and the mechanism of elimination of these carriers. Another important parameter to be considered is the dose of anticancer drug and cyclodextrin used in the formulation. The extraordinary features of both these systems (cyclodextrin and nanotechnology) will simultaneously offer additional avenues to treat cancer successfully. Furthermore, there is a prerequisite to exploit the utility of these cyclodextrin-based nanocarriers using* in vivo* models for tumor targeting and toxicity studies of cancer like life-threatening disease. In future, cyclodextrins could be employed for modification of potent anticancer drugs to achieve effective treatment.

## Figures and Tables

**Table 1:** Characteristics features of different types of cyclodextrins.

Name of cyclodextrin	Solubility (mg/mL)	Mol. Wt. (Da)
Natural cyclodextrins		
Alpha cyclodextrin	145	972
Beta cyclodextrin	18.5	1135
Gamma cyclodextrin	232	1297

Chemically modified cyclodextrins		
Hydroxypropyl-*β*-cyclodextrin	≥600	1400
Sulfobutyl ether-*β*-cyclodextrin	≥500	2163
Randomly methylated-*β*-cyclodextrin	≥500	1312
Hydroxypropyl-*γ*-cyclodextrin	≥500	1576

Polymerized cyclodextrins		
Epichlorohydrin-*β*-cyclodextrin	>500	112000
Carboxy methyl epichlorohydrin beta cyclodextrin	>250	2000000–15000000

**Table 2:** Complexation of various anticancer drugs with cyclodextrin and their derivatives.

Serial number	Drug	Use	Cyclodextrin	Method	Outcome	Reference
1	9-Nitro camptothecin	Pancreatic cancer	HP-*β*-CD	Colyophilization	Significant improvement in antitumor activity and reduction in toxicity	[45]

2	Methotrexate	Melanoma	*β*-CD HP *β*-CD	Neutralization	Enhancement of aqueous solubility and bioavailability	[46]

3	Lonidamine	Prostate cancer	PM-*β*-CD	Physical mixture	Enhancement of solubility	[47]

4	Exemestane	Breast cancer	M *β*-CD	Kneading	Increase in solubility, improvement in bioavailability and dissolution	[48]

5	Vorinostat	Lymphoma	HP-*β*-CD, RM *β*-CD	Freeze-drying	Enhancement of bioavailability	[49]

6	Imatinib	Chronic leukemia	*β*-CD, RM *β*-CD	Freeze-drying	Enhancement of solubility	[50]

7	Doxorubicin	Lymphoma and leukemia	HP *β*-CD	Freeze-drying	Stability to acid hydrolysis and photodegradation	[51]

8	Cisplatin	testicular, ovarian, and cervical carcinoma	HP-*β*-CD	Freeze-drying and physical mixture	Increase in solubility, improvement in dissolution rate, and reduction of toxicity	[52]

9	Flutamide	Prostatic carcinoma	*β*-CD, HP-*β*-CD	Lyophilization	Enhancement of solubility and dissolution	[53]

10	Zerumbone	Colon and skin cancer	HP-*β*-CD	Freeze-drying	Improvement in solubility, stability, and bioavailability	[54]

11	Melphalan	Multiple myeloma and ovarian cancer	HP-*β*-CD	Freeze-drying	Stability against hydrolysis, solubility enhancement	[55]

12	Oridonin	Esophageal and cardiac cancer	*β*-CD	Freeze-drying	Enhancement of bioavailability	[56]

13	5-Fluorouracil	Cervical cancer	*β*-CD, HP *β*-CD	Colyophilization	Enhancement of solubility	[57]

**Table 3:** Cyclodextrin-based carriers of anticancer drugs.

Serial number	Drug	Cyclodextrin	Nanocarrier prepared	Outcome	Reference
1	Doxorubicin	*γ*-CD	Liposomes	Increased retention in tumor cells	[59]
2	Curcumin	HP-*γ*-CD	Liposomes	Improvement in therapeutic efficacy	[60]
3	Methotrexate	*β*-CD	Niosomes	Increased entrapment efficiency and solubility	[61]
4	Camptothecin	*β*-CD	Nanosponges	Improvement in therapeutic efficacy and reduction in toxic effects	[62]
5	Paclitaxel	*β*-CD	Nanosponges	Prolonged shelf life	[63]
6	Doxorubicin	*β*-cyclodextrin-based star copolymers	Micelles	Enhanced drug release	[64]
7	*β*-lapachone	*α*-CD	Polymeric millirods	Sustained drug release	[65]
8	Camptothecin	Amphiphilic *β*-CD	Nanoparticles	Prolonged drug release	[66]
9	Paclitaxel	Amphiphilic *β*-CD	Nanoparticles	Increased drug loading capacity	[67]
10	Docetaxel	Amphiphilic *β*-CD	Nanoparticles	Increased solubility and prolonged release	[68]
